# Nutritional value of raw *Canavalia ensiformis* and its utilization as partial replacement for soybean meal in the diet of *Clarias gariepinus* (Burchell, 1822) fingerlings

**DOI:** 10.1002/fsn3.548

**Published:** 2017-11-12

**Authors:** Shola G. Solomon, Victor T. Okomoda, Obekpa Oguche

**Affiliations:** ^1^ Department of Fisheries and Aquaculture University of Agriculture Makurdi Nigeria

**Keywords:** African catfish, EAAs, jack bean, nutrient utilization, unconventional feeds

## Abstract

The nutritional value of raw Jack bean meal (*Canavalia ensiformis*) as a partial substitute for soybeans meal was investigated in this study. Preliminary investigation on nutrient composition revealed that lysine, histidine, and phenylalanine were significantly higher in *C*. *ensiformis* seed meal compared to soybean meal. However, crude protein and other essential amino acids were significantly lower. Feeding trial was then conducted to investigate the effect of replacing about 40% soybeans meal (at 58.8% inclusion) with *C*. *ensiformis* in the diet of *Clarias gariepinus*. The result obtained after 56 days revealed that fingerlings could tolerate up to 20% replacement without significant effect on growth and nutrient utilization. Beyond this, growth was significantly reduced. Survivals of the fish also follow a similar trend as stated above. It was concluded that dietary inclusion of raw *C*. *ensiform* meal should not be beyond 11% (or 20% replacement for soybeans meal included at 58.8%) in the diet of *C*. *gariepinus*.

## 1 INTRODUCTION

Attention towards aquaculture in the last decade has been mainly due to increased demands for aquatic products (Allan, 2004) and continuous decrease in natural fish production (Solomon, Okomoda, & Aladi, 2012). The resultant effect of the continuous decrease in wild fish stock is increased scarcity and hike in prices of the aquatic product. Aquaculture hence presents the best ways to cope with this problem aside effort channeled toward stewardship of natural fisheries resources and development (CGIAR, 2005). The survival and well‐being of any confined animal are highly dependent on the availability of balanced food materials (Umar, 2013). Hence, rearing species under aquaculture require an input of nutrients for growth, reproduction, development, and tissue maintenance. Fish, especially when reared in high density or confined in cages cannot forage freely on natural foods, hence, requires a high quality, nutritionally complete balanced diet to grow rapidly and remain healthy (Houlihan, Bouiard, & Jobling, 2001).

It is estimated that nutrition in fish farming is about 40%–60% of the total production cost of the fish (Craig & Helfrich, 2002; Jamiu & Ayinla, 2003; Steven, 2001; Tiamiyu, Solomon, & Sham, 2008). Hence, it could be rightly said that nutritional management largely determines the profitability or loss in a fish farming venture. The conventional feedstuffs used in feed formulation are continuously increasing in price because of competitive need from other sectors as well as human consumption (Tiamiyu, Solomon, & Satimehin, 2014). Therefore, to reduce the cost of feeding and providing steady feed supply to cultured species, unconventional feedstuff are currently identified and researched for their suitability in the nutrition of most aquaculture candidate (Obasuyi & Nwokoro, 2005).

Legumes have been an important crop ever since the domestication of plants began (Crespo, 1987). Many legumes possess multiple uses as food, fodder, and pharmaceutical (Sridhar & Bhat, 2007). A wide variety of legumes has been accessed as alternatives to fishmeal most of which are of limited relevance in human nutrition (Osuigwe, 2003). Research has also shown that they represent good dietary protein and energy sources for fish (Jauncey, 1998). The genus Canavalia (Jack bean) comprises 48 species of underutilized legumes indigenous and widely distributed in the tropics (Tiamiyu, Okomoda, & Akpa, 2016). It is rarely eaten by man and has a total yield of up to 2.5 tons·ha^−1^ under optimal agronomic conditions (Okonkwo & Udedibie, 1991). Raw *Canavalia ensiformis* seeds contain about 300 g·kg^−1^ protein and 600 g·kg^−1^ carbohydrates (Rajaram & Janardhanan, 1992), hence, they have great potential as a replacement for conventional feedstuffs. The aim of this study is, therefore, to first, advance knowledge on nutritional qualities of *C*. *ensiformis* by determining their proximate and essential amino acid content compared to conventional soybeans. Secondly, we determine the utilization of the feeds formulated with partially substituted levels of soybeans for *C*. *ensiformis* seeds meal in the diet of African catfish *Clarias gariepinus*.

## 2 MATERIALS AND METHODS

### 2.1 Feed procurement, processing, and nutritional analysis

Matured seeds of *C. ensiformis* were obtained from Assakio in Lafia Nasarawa State Nigeria. The seeds were cleaned, milled, and stored in a dry place. Other ingredients for the feed formulation such as soybeans meal, maize meal, fish meal, vitamin, and mineral premises were purchased from a feed store in the modern market. Samples of the *C. ensiformis* seed meal were sent to the Department of Fisheries and Hydrological Laboratory, in the University of Jos for nutritional analysis of proximate and essential amino acid profile. The proximate composition of the seed meal was determinate using standard methods according to AOAC (2000). The amino acid profile, however, was determined using the method described by Spackman, Stein, and Moore (1958). The feeding trial for this study was done at the Department of Fisheries and Aquaculture Research Farm, University of Agriculture Makurdi, Benue state Nigeria.

The soybeans purchased were toasted at 100°C for 30 min to inactivate the anti‐nutritive factors present following the method of Tiamiyu et al. (2016). Toasting was done by continuously stirring the seeds in fine texture sand heated on a hot plate. This is to ensure uniformity and prevents charring of the seed by the heat. The toasted seeds were then grounded into a meal and stored in separate packaging materials in a cool, dry place for the diets formulation.

### 2.2 Diet formulation

Fully aware of the fact that soybean contains more crude protein (44%CP) compared to Jack bean meal (25%CP), partial replacement feeding trial was designed to elucidate the nutritional suitability of the feedstuff in the diet of *C*. *gariepinus*. A control diet (Diet 1) was formulated having 35% crude protein (Table 1) while four subsequent experimental diets had soybean content of the meal replaced by *C. ensiformis* seed meal at 10%, 20%, 30%, and 40% (respectively denoted as Diet 2, Diet 3, Diet 4, and Diet 5). All ingredients were sieved, weighed, and mixed uniformly. Hot water at 60°C was added to the mixture and stirred to form a dough. The dough was pelleted using a 2 mm‐die and the resulting pellets sun‐dried for 3 days. The diets were packaged and stored for use.

**Table 1:** Gross composition (g·kg^−1^) of diet containing partial replacement of soybean meal with *C. ensiformis* meal

Ingredients	Diet 1	Diet 2	Diet 3	Diet 4	Diet 5
Maize meal	261.8	261.8	261.8	261.8	261.8
Soybeans meal	588.2	529.38	470.56	411.74	352.92
Fish meal	100	100	100	100	100
Jack bean meal	0	58.82	117.64	176.46	235.28
Vitamin premix	20	20	20	20	20
Mineral premix	20	20	20	20	20
Salt	5	5	5	5	5
Oil	5	5	5	5	5

### 2.3 Experimental setup, fish performance evaluation, and data analysis

One thousand fingerlings of *C*. *gariepinus* were obtained from the University Fish Farm and acclimatized for 2 weeks before the start of the feeding trials. During the acclimatization period, fish were fed Coppen commercial diet (45% CP). Fifteen hapas (for the five treatments with three replicates) measuring 1 × 1 × 1 m^3^ were mounted on two‐kuralon ropes and set across a 45 × 45 × 2 m^3^ earthen pond. The ropes were properly staked to the dyke of the pond using bamboo sticks. Metal sinkers were attached to the four bottom corners of each hapa to ensure uniform spread and proper extension. This is to allow easy inflow and outflow of water through each hapa system. The system was set in such a way that hapas were submerged half way below the water level to enable easy access to the fish. Hapas were labeled in triplicate according to the five experimental diets to be administered. Fifteen batches of 50 fingerlings (0.78 ± 0.03 g) were weighed and stocked randomly in each of the fifteen hapas and fed the experimental diets for 56 days. After which growth and nutrient utilization were assessed using the relations shown below;

- Weight gain (g) = *W*_2_−*W*_1_
- Growth rate (g/d) = $\frac{W_{2}-W_{1}}{t_{2}-t_{1}}$
- Specific growth rate (%/day) = $\frac{{\text{log}}_{e}\left(W_{2}\right)-{\text{log}}_{e}\left(W_{1}\right)}{t_{2}-t_{1}}$Where *W*_1_ = initial weight (g)*W*_2_ = final weight (g)*t*_2_−*t*_1_ = duration between *W*_2_ and *W*_1_(d)
- Feed conversion ratio (FCR) = $\frac{\text{dryfeed intake}}{W_{2}-W_{1}}$
- Feed efficiency ratio (%FER) = $\frac{(W_{2}-W_{1})\;\;\times \;\;100}{\text{dryfeed intake}}$
- Protein efficiency ratio = $\frac{W_{2}-W_{1}}{\text{protein fed}}$Where protein fed = $\frac{\%\text{protein in diet}\;\times \;\text{total diet consumed}}{100}$
- %Survival rate = $\frac{\text{fish stocked}-\text{mortality}}{\text{fish stocked}}\times 100$

Pond water quality was maintained by the addition of fresh river water from the River Benue on a daily basis through a network of pipes. Estimated daily water replacement in the pond was about 20%. Water quality parameters such as temperature (26.1 ± 1.5), pH (7.53 ± 0.05), conductivity (543 ± 2.5), total dissolved solids (271.5 ± 6.0), and dissolved oxygen (5.6 ± 0.5) concentration were monitored weekly in the ponds using a digital multi‐parameter water checker (Hanna water tester Model HL 98126). Proximate compositions of the formulated diet, carcass of fish before and after the feeding trial were also determined using the official method by AOAC (2000). Summary statistics of the different variables measured across the treatment were obtained using Minitab 14 for Windows software (McKenzie, 2004). The result of the nutritional profile of jack bean and soybean were compared using student *T* test, while the result of the nutritional trial was subjected to Analysis of Variance (ANOVA). Where significant differences occurred; means were separated using Fisher's least significant difference.

## 3 RESULT AND DISCUSSION

### 3.1 Proximate composition of raw *C*. *ensiformis* and Soybean

The moisture and ash contents of raw *C. ensiformis* meal (7.2% and 3.9%, respectively) are within the range expected of most legumes (Olaofe & Sanni, 1988; Oyenuga, 1968). Ash content of raw *C*. *ensiformis* meal reported in this study is higher than values reported by Saulawa, Yaradua, and Shuaibu (2014) for soybean but lower the value recorded for soybean in this study. The nutritional content of plant is variable and depend on the strain, environmental factors, and processing method. Hence, the differences observed between laboratory report of this study for soybean and the cited literature. The crude protein of *C. ensiformis* in this study (Table 2) is higher than that reported for pigeon pea (Onimawo & Osugo, 2004), cowpea and bambara (Nwokolo & Oji, 1985). The fat content of the raw *C. ensiformis* meal (5.2%) is lower than that observed in soybeans meal (7.0%). The high‐fat content of feedstuff is theoretically expected to be a good source of energy and also reduce dustiness in feeds. This is likely to influence better utilization of protein for growth rather than being used for energy supply (Tiamiyu et al., 2014).

**Table 2:** Proximate composition (%) of *Canavalia ensiformis* and soybeans seed

Parameters	*C. ensiformis*	Soybeans	SEM
Moisture	7.24^a^	7.07^b^	0.24
Ash	3.88^b^	5.87^a^	1.03
Lipid	5.25^a^	7.03^b^	0.34
Fibre	7.14^a^	4.38^b^	0.09
Protein	25.31^b^	43.80^a^	2.40
NFE	51.36^b^	31.82^a^	3.02

NFE, nitrogen free extract.

Numbers are means. Mean in the same row with different superscripts differ significantly (ANOVA, *p* ≤ .05)**.**

### 3.2 Essential amino acid profile of raw *C*. *ensiformis* and Soybean

In general, three amino acid namely histidine, phenylalanine, and lysine were significantly higher (Table 3) in *C. ensiformis* meal (2.6, 5.2, and 6.5, respectively) compared to the value recorded in soybean meal (2.3, 4.1, and 6.4, respectively). Valine and tyrosine in the raw *C. ensiformis* meal are higher in value when compared with values reported for pigeon pea (Akande, Doma, Agu, & Adamu, 2010; Apata & Ologhobo, 1994). Leucine in the raw seeds is lesser than the value reported for soybeans in this study but higher than values reported by Temple and Aliyu (1994) for soybean. The ratio of leucine and isoleucine for raw *C. ensiformis* meal (7.1: 3.2) observed in this study is higher in value but similar in ratio to those reported in the fishmeal (4.5:2.9, respectively). The leucine/isoleucine ratio is a major factor limiting inclusions of conventional and unconventional feedstuffs at a higher level in the diet of fish (Tiamiyu, Okomoda, & Iber, 2013). Feed ingredient that is greater in leucine but lower in isoleucine result in antagonism between leucine and isoleucine, hence, leading to acute deficiency of isoleucine at higher inclusion level (Crawshaw, 1994). Lysine is an essential amino acid in animal feed and usually deficient in cereal grain‐based diets for fish, resulting in reduced growth (Cheng, Hardy, & Usry, 2003; Mostafa, Rahma, & Redy, 1987). The concentration of lysine reported in this study for raw *C. ensiformis* meal makes it a useful substitute for cereal grain. Raw *C. ensiformis* meal was found to be deficient in the sulfur‐containing amino acids such as methionine (1.05) and cystine (1.10) compared to the values recorded in soybeans meal (1.42 and 1.68, respectively). However, values recorded correlate with the values reported for pigeon pea by Akande et al. (2010). Generally, low concentrations of methionine and cystine have been reported in many other legumes in previous research (Adeyeye & Afolabi, 2004; Apata & Ologhobo, 1990; Aremu, Olaofe, & Akintayo, 2006; Doku, Hammonds, & Francis, 1978). Jansman (1996) concluded that lysine constituted the highest amino acids in many legumes while sulfur amino (methionine and cystine) acids are most lacking.

**Table 3:** Essential amino acid composition (g/100 g protein) of *Canavalia ensiformis* and soybeans seed

Parameters	*C. ensiformis*	Soybeans	SEM
Lysine	6.55^a^	6.41^b^	0.21
Histidine	2.64^a^	2.43^b^	0.02
Methionine	1.05^b^	1.42^a^	0.17
Threonine	3.44^b^	3.91^a^	0.03
Isoleucine	3.25^b^	4.64^a^	0.54
Leucine	7.03^b^	7.78^a^	0.35
Tyrosine	3.12^b^	4.21^a^	0.50
Valine	4.21^b^	4.61^a^	0.06
Phenylalanine	5.24^a^	4.15^b^	0.23
Cystine	1.10^b^	1.68^a^	0.04

Numbers are means. Mean in the same row with different superscripts differ significantly (ANOVA, *p* ≤ .05).

### 3.3 Growth performance and nutrient utilization by *C*. *gariepinus* fed substituted levels of *C*. *ensiformis* seed meal

The dietary inclusions of raw *C*. *ensiformis* led to significant reduction in dietary protein (Table 4) as the substitution levels increased (35.16% in Diet 1 and 34.56% in Diet 2) as observed in this study. This was expected and presumed to be as a result of the lower protein content of raw *C*. *ensiformis* meal (25%CP) compared to soybean meal (44%CP) (Table 2). Though the dietary fiber content increased as the level of inclusion of raw *C. ensiformis* meal increased, they were still within recommended ranges as reported by Gatlin (2010). Gatlin (2010) also stated that fiber content below 7% helps reduce the number of undigested materials entering the culture system. The result of the feeding trial of dietary substitution of soybean for raw *C*. *ensiformis* meal reveals that *C*. *gariepinus* can tolerate up to 20% substitution with raw *C*. *ensiformis* meal which is 11.8% level of inclusion in the diet. Beyond this point, growth and nutrient utilization significantly reduced compared to the control (Table 5). This may be as a result of nutrition reduction in protein and fat as the level of inclusion increased in the diet. Craig and Helfrich (2002) earlier pointed out that protein is used for growth if adequate levels of fat and carbohydrates are present in the diet. If not, the protein may be used for energy and life support rather than growth. Furthermore, Steffens (1993) concluded that increasing dietary lipid with high‐quality fats improves growth, feed conversion, protein utilization, and reducing nitrogen excretion. It can then be said that dietary reduction in protein and fat as a result of inclusions of the raw *C*. *ensiformis* meal beyond 20% made the diet nutritionally inferior compared to the inclusions below 20%. Aside from this stated fact of the nutritional inferiority of the diets, observed reduced growth performance beyond 20% substitution could be as a result of elevated levels of anti‐nutritional factors (ANFs) in the raw *C. ensiformis* meal beyond the tolerant level of the fish. Although ANFs were not reported in this study, Martinez‐Palacios, Galvan, Olvera‐Novoa, and Charvez‐Martinez (1998), Akinbiyi (1992) and Abdo de la Parra et al. (1998) had earlier stated that *C*. *ensiformis* seed meals utilization by fish is basically limited by the presence of anti‐nutritional factors. Some of these ANFs have been identified to be thermostable in nature and could result in lethargic behavior, poor growth, and mortality of the fish (Okomoda, Tiamiyu, & Uma, 2016). Hence, this could have been the reason for the low survival (<77%) observed in the diet with *C*. *ensiformis* beyond 20% substitution for soybeans (Table 5). The result of this study compared to the report by Martinez‐Palacios et al. (1998), Akinbiyi (1992), and Abdo de la Parra et al. (1998) shows that *C*. *gariepinus* can tolerate higher inclusion of raw *C*. *ensiformis* mean (11% level of inclusion) better than *O*. *niloticus* (5% level of inclusion). However, Fagbenro, Adeparusi, and Jimoh (2004) reported improvement of growth, nutrient utilization, and bioavailability of *O*. *niloticus* with dietary inclusion of 20% cracked jack bean seeds cooked in distilled water and also at 30% inclusion level of cooked jack bean seeds in trona solution. While Okomoda et al., 2016 reported improvement in the performance of *C*. *gariepinus* fed 27% inclusion levels of hydrothermally processed *C*. *ensiformis* seeds (up to 40 min). These studies suggest that with appropriate processing methods, dietary inclusions in African catfish can be utilized beyond the level reported in this study.

**Table 4:** Proximate composition (% of dry matter) of the diets fed to *Clarias gariepinus* fingerlings

Parameters	Diet 1	Diet 2	Diet 3	Diet 4	Diet 5	SEM
Moisture	8.36^c^	8.31^d^	9.42^a^	8.57^b^	7.61^e^	1.02
Ash	8.36^c^	8.31^d^	9.41^a^	7.57^b^	8.04^e^	0.42
Fat	5.77^a^	5.65^b^	5.45^c^	5.43^c^	5.33^d^	0.55
Fibre	6.38^c^	5.94^e^	6.44^b^	6.55^a^	6.33^d^	0.12
Protein	35.16^a^	35.08^b^	34.84^c^	34.77^d^	34.56^e^	0.02
NFE	35.54^d^	35.92^c^	34.53^d^	36.11^b^	37.04^a^	1.03

NFE, nitrogen free extract.

Numbers are means. Mean in the same row with different superscripts differ significantly (ANOVA, *p *≤* *.05).

**Table 5:** Nutritional indices of *Clarias gariepinus* (initial weight of 0.78 ± 0.03) fed diets containing partially replaced soybeans with *Canavalia ensiformis* meal for 56 days

Parameters	Diet 1	Diet 2	Diet 3	Diet 4	Diet 5	SEM
Final weight (g)	3.81^a^	3.78^a^	3.53^a^	2.66^b^	2.69^b^	0.45
Weight gain (g)	3.03^a^	2.98^ab^	2.75^b^	1.89^c^	1.89^c^	0.23
Growth rate (g·day^−1^)	0.054^a^	0.052^a^	0.050^a^	0.034^b^	0.034^a^	0.02
SGR (%/day)	2.82	2.74	2.71	2.29	2.27	0.31
FCR	2.06^c^	2.19^b^	2.21^b^	2.81^a^	2.76^a^	0.32
FER (%)	44.24^a^	43.08^a^	42.05^a^	36.00^b^	37.46^b^	2.04
PER	0.087	0.071	0.064	0.054	0.054	0.06
Survival (%)	96.67^a^	95.3^a^	94.70^a^	76.70^b^	66.70^c^	2.10

SGR, specific growth rate, FCR, feed conversion ratio, PER, protein efficiency ratio.

Numbers are means. Mean in the same row with different superscripts differ significantly (ANOVA, *p *≤* *.05).

The trend for carcass composition in this study agreed with the hypothesis of Abbas (2007) and Manjappa, Keshavanath, and Gangadhara (2011). They hypothesize that the level of nutrient utilization and retention in the carcass of fish fed experimental diet is related to both the dietary protein levels and the availability of non‐protein energy sources with the lower inclusion of dietary fiber. Hence, higher carcass protein was discovered in fish fed higher dietary protein as a result of the lesser inclusion of raw *C*. *ensiformis* meal and vice versa (Table 6). The result of this study is also similar to the findings of Keembiyehetty and De Silva (1993) for cowpea (*Vigna catiang*) and black gram (*Phaseolus mungo*) seeds fed to *O. niloticus*. Also, similar to the report of Olvera‐Novoa, Martinez, Galvan, and Chavex (1988) and Martinez‐Palacios et al. (1998) who fed Sesbania grandiflora and jack bean seed meal, respectively to *O*. *mossambicu*s.

**Table 6:** The proximate composition (% of dry matter) of carcass of fingerlings of *Clarias gariepinus* fed diets containing partially replaced soybeans with *Canavalia ensiformis* meal

Parameters	Initial	Diet 1	Diet 2	Diet 3	Diet 4	Diet 5	SEM
Moisture	3.22^d^	3.45^b^	3.45^b^	3.57^a^	3.35^c^	3.37^c^	2.34
Ash	3.07	3.33	3.11	3.27	3.35	3.59	0.43
Fat	4.46^d^	5.13^a^	5.11^a^	4.87^b^	4.83^b^	4.59^c^	1.92
Fibre	3.03^c^	3.22^a^	3.11^b^	3.12^b^	3.21^a^	3.15^b^	2.01
Protein	47.94^f^	50.77^a^	50.21^ab^	50.14^b^	49.77^c^	49.55^c^	1.31
NFE	38.294^a^	34.13^e^	35.03^d^	35.05^d^	35.51^c^	35.75^b^	0.92

NFE, nitrogen free extract.

Mean numbers are means. Mean in the same row with different superscripts differ significantly (ANOVA, *p *≤* *.05).

## 4 CONCLUSIONS

This study demonstrated that raw *C*. *ensiformis* can be utilized up to 11% inclusion level (corresponding to 20% replacement for soybeans meal at 58.8% inclusion) without adversely affecting the growth of the fish. Improving nutritional value of *C*. *ensiformis* using conventional processing method should be the focus of subsequent research. The outcome of these researches will be beneficial not just for commercial industries but for on‐farm production of animal feeds as it will reduce the cost of production.

## CONFLICT OF INTEREST

None declared.
